# Using Computer Vision to Annotate Video-Recoded Direct Observation of Physical Behavior

**DOI:** 10.3390/s24072359

**Published:** 2024-04-08

**Authors:** Sarah K. Keadle, Skylar Eglowski, Katie Ylarregui, Scott J. Strath, Julian Martinez, Alex Dekhtyar, Vadim Kagan

**Affiliations:** 1 Department of Kinesiology and Public Health, California Polytechnic State University, San Luis Obispo, CA 93407, USA; kylarreg@calpoly.edu; 2 Sentimetrix Inc., Bethesda, MD 20814, USA; skylar@sentimetrix.com (S.E.); kagan@sentimetrix.com (V.K.); 3 College of Public Health, University of Wisconsin, Milwaukee, WI 53205, USA; sstrath@uwm.edu (S.J.S.); marti994@uwm.edu (J.M.); 4 Department of Computer Science and Software Engineering, California Polytechnic State University, San Luis Obispo, CA 93407, USA; dekhtyar@calpoly.edu

**Keywords:** physical activity, sedentary behavior, assessment, direct observation, computer vision

## Abstract

Direct observation is a ground-truth measure for physical behavior, but the high cost limits widespread use. The purpose of this study was to develop and test machine learning methods to recognize aspects of physical behavior and location from videos of human movement: Adults (N = 26, aged 18–59 y) were recorded in their natural environment for two, 2- to 3-h sessions. Trained research assistants annotated videos using commercially available software including the following taxonomies: (1) sedentary versus non-sedentary (two classes); (2) activity type (four classes: sedentary, walking, running, and mixed movement); and (3) activity intensity (four classes: sedentary, light, moderate, and vigorous). Four machine learning approaches were trained and evaluated for each taxonomy. Models were trained on 80% of the videos, validated on 10%, and final accuracy is reported on the remaining 10% of the videos not used in training. Overall accuracy was as follows: 87.4% for Taxonomy 1, 63.1% for Taxonomy 2, and 68.6% for Taxonomy 3. This study shows it is possible to use computer vision to annotate aspects of physical behavior, speeding up the time and reducing labor required for direct observation. Future research should test these machine learning models on larger, independent datasets and take advantage of analysis of video fragments, rather than individual still images.

## 1. Introduction

The numerous health benefits of a physically active lifestyle are well-established, and the implementation of devices to measure physical behavior in prospective cohorts and randomized clinical trials has provided important new insight into the type and amount of physical activity that is needed for optimal health benefits [1,2,3]. However, there is heterogeneity in data collection procedures and data processing methods (i.e., statistical algorithms applied to device signals to estimate aspects of physical behavior, such as time spent in moderate-intensity physical activity), which causes a great deal of confusion in the field as different studies are reaching different conclusions about the amount of physical activity for optimal health benefits [4,5,6]. In order for device data to provide consistent, accurate estimates of physical behavior, there is a need for rigorous validation studies that include data collection in a large number of participants, within naturalistic conditions and established ground-truth measures [7].

Video-recorded direct observation, which includes frame-by-frame analysis of a participant, is a ground-truth measure that can be used to validate many physical behavior metrics that are linked to health, including steps, postural transitions, behavior type (e.g., housework, walking) and location (e.g., work, park) [8,9,10,11,12]. However, collecting and analyzing this ground-truth method is often prohibitively expensive due to the time, costs, and training required to manually annotate images, making its utility in large studies impractical [13]. Recent advances in machine learning technology as applied to computer vision have demonstrated the potential of automating image annotation. Using multi-layered special purpose neural networks (convolutional neural networks, recurrent neural networks), researchers have been able to accurately classify images based on what is depicted in them, recognize the position of objects of interest in an image, recognize humans in an image, and track objects (vehicles, humans) across multiple consecutive frames of a video [14,15,16].

Datasets for recognizing humans and for classifying on-screen human actions and activities abound, as does research on human action detection. Herath et al. [17] note in their survey of work on automated recognition of human activities that researchers interpret the notion of “action” to be detected in drastically different ways: from capturing atomic limb movements [18,19] to recognizing “simple motion patterns … lasting for a very short duration (order of tens of seconds)” [20] to attempting to build a hierarchical representation of the notion of “action” [21]. Yet, there is a difference between these varied views on what constitutes an action and the notion of physical behavior used in public health research, which are based on constructs and taxonomies that are defined by the American Time Use Survey, Compendium of Physical Activities and the Sedentary Behavior Research Network Consensus Terminology Project [22,23,24]. To our knowledge, none of the widely used datasets in computer vision research contain ground-truth annotations compatible with the standardized terminology in the fields of physical behavior and health, nor do they contain video footage taken during human activity studies in naturalistic conditions; nor have there been any open-source models trained on such taxonomies.

To date, the primary application of this computer vision to physical activity and health studies has focused on quantifying aspects of the built environment that may affect physical activity levels [25,26,27,28]. Carlson et al. demonstrated the utility of computer vision for evaluating aspects of the physical environment, including the number of people in a park and identifying how many were engaging in physical activity [26]. This work primarily has used two types of video or still image data—a first-person (or “ego-centric”) perspective where the images reflect what the participant can see externally but do not contain their body within the image or a fixed camera where the camera stays in the same place, but people come in and out of view [26,29,30]. Limited work has been done using third-person images, where the full body of a single participant is shown continuously. The third-person point of view is particularly beneficial when video-recorded direct observation provides ground truth for device algorithm development because the participant’s whole body can be seen, enabling detailed annotation of body posture transitions and activity types and locations within their natural environment [13].

The primary aim of this study was to develop and test machine learning methods that estimate physical behavior metrics from video-recorded direct observation that includes a single participant for a 2-h period. Specifically, we examined the following taxonomies that are aligned with consensus labels in physical activity and health research: (1) sedentary versus not; (2) general activity type (sedentary, mixed movement, walking and running); and (3) activity intensity (sedentary, light, moderate, and vigorous) [22,24].

## 2. Materials and Methods

Participants were recruited from San Luis Obispo, CA, and the surrounding communities through word of mouth and fliers. All participants read and signed an Informed Consent Document approved by the Cal Poly Institutional Review Board. Participants were aged between 18 and 59 years old and were generally healthy with no major orthopedic injuries that inhibited their ability to perform exercise and/or walk. Information including age (yrs), sex, race/ethnicity, height and weight, and physical activity status (response options range from 0—avoided walking or exertion to 7- ran more than 10 miles per week or sport over 3 h per week in comparable activity) were assessed at the first study visit. In total, 26 participants consented and were enrolled with an average mean (SD) of 30.5 (11.5) years old and an average BMI of 24.6 (3.8) mg/m^2^; 63% of participants were female.

Participants were scheduled for two, 2-h video-recorded direct observation (DO) sessions. Participants were recorded using a GoPro (GoPro, Inc., San Mateo, CA, USA) Hero 5 camera. Two observers were present during each session. The primary observer held the Hero 5 and the secondary observer recorded details of the DO session. Observers were trained to avoid interacting with or influencing the participant’s behavior. For each session, participants were instructed to complete their normal daily activities. Each DO session was assigned one of five time-use-based categories to ensure a wide range of activities and settings were observed. The categories and general constraints were as follows: (1) work sessions took place in the participant’s typical work setting. If their occupation was sedentary office work, they were instructed to take at least two breaks from their desk during the two-hour period; (2) household sessions took place in a participant’s house, and they were instructed that at least 45 min of the session should be household or personal care activities such as meal preparation, gardening, house cleaning, caring for others, laundry, and/or dishwashing; (3) sedentary leisure consisted of the participant being seated and/or lying on furniture while performing a leisure activity such as watching television, playing a video game, and/or reading a book. They were instructed to take at least one break from sitting; (4) active leisure sessions consisted of participants spending at least 45 min in a leisure-time physical activity (e.g., walking, weightlifting, running, playing frisbee, sports, and hiking); and (5) community sessions consisted of observing a participant taking some form of transportation (e.g., bike, bus, car) from their initial environment to a new environment located in a community setting such as a grocery store, a sports stadium, a restaurant, or a local venue. The participant must have a reason for going to the new environment, such as buying groceries, meeting up with friends, or attending an event.

### 2.1. Direct Observation Annotation 

When downloaded, the GoPro files were spliced into 20-min clips, which were edited into a single video using Adobe Premiere (Adobe, Inc., San Jose, CA, USA). The videos were annotated using the Noldus Observer XT 14 program (Noldus Information Technology, Wageningen, The Netherlands). Research assistants were trained using established protocols adapted for the use of video recording [11,13,31]. All assistants read a written manual and then completed 12 h of practice coding. Observers then coded two practice videos that included a range of behaviors, postures, and intensities using the full coding scheme and were required to obtain an intraclass correlation (ICC) >0.9. To score the actual study data, the coders used a multi-pass method. In the first pass, they coded for behavioral domains consistent with the American Time Use Survey [23]. In the second pass, observers coded for posture/movement (lying down, sitting/reclining, kneeling/squatting, stretching, standing, stand and move, walk, walk with load, running, biking, ascending stairs, descending stairs, muscle strengthening activities, and sport movement) and intensity (sedentary, light, moderate, and vigorous). The posture coding scheme was designed to align with the consensus taxonomy on physical behaviors, and intensity was defined using the Compendium of Physical Activity and SBRN Consensus Terminology Project as a reference [22,24]. A random sample of 20% of the videos were dual-coded by all coding staff, with high inter-rated reliability (ICC = 0.95).

**Figure 1:**
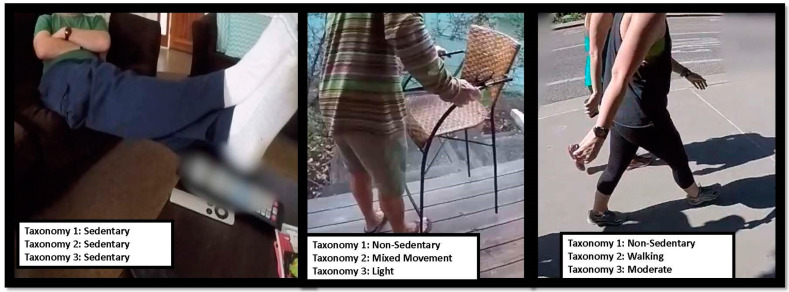
Sample images from videos with corresponding labels by taxonomy. Note: Taxonomy 1 is sedentary or not, Taxonomy 2 is activity type (four classes), and Taxonomy 3 is activity intensity (four classes).

The annotated data were exported from Noldus into Python (version 3.5.4) in event-based format and converted to second-by-second estimates. If multiple behaviors or postures occurred within the same second, these were labeled as transitions, and the behavior/posture that occurred for >50% of the second was assigned as the primary behavior/posture. For this initial proof of concept, the full annotation schemes were consolidated into the following taxonomies, and we trained and evaluated separate models for each of the taxonomies (See Figure 1).

1. Sedentary versus non-sedentary (two classes): Sedentary was considered sitting or lying, and all other postures/whole-body movements were non-sedentary.
2. Activity type (four classes): Sedentary was defined the same as Taxonomy 1, with the non-sedentary broken up into three new categories: walking, running, and mixed movement. Mixed movement covers all other postures/whole-body movements.
3. Activity intensity (four classes): Sedentary is low energy expenditure while seated, light is standing and <3 metabolic equivalents (METs), Moderate is 3–5.99 METs, and Vigorous is greater than or equal to 6 METS) [24,32].

### 2.2. Image Data Processing 

Our dataset was composed of approximately 5000 min (81.5 h) of annotated activity filmed in 43 different videos. For each second of annotated video, we extracted a single representative frame for a total of 293,700 images. Videos are recorded at high resolution and fps (1080p with 30 fps) and then downscaled to 2 fps to align with ground truth. To fit the specific demanded resolution from our pre-trained models, either 224 × 224 or 384 × 384, we downscaled the longest edge to the desired resolution, keeping the aspect ratio intact. We then padded in the excess space with black pixels. This enabled the handling of a diverse number of orientations and input resolutions.

We then placed each input video into one of three categories: training, testing, or evaluation. These groups are referred to as “folds”. The training fold constituted 80% of the data and was used to train the machine learning models. The testing fold constitutes 10% of the data. This set served multiple purposes related to the regular assessment of intermediate or prototype versions of each model. For example, the testing fold was used to help address “overfitting”, which occurs when the model begins to form overly specific assumptions about incidental correlations in the training data that do not generalize well to unseen data. By periodically evaluating a deep learning model during its training, we can pinpoint an inflection point where the reported accuracy of the training fold stops increasing and begins to decrease. Once this trend is consistent, further training stops and reverts the model to the state it was at the inflection point. The third category is evaluation, which also made up 10% of the data. This data was held in reserve until, after sufficient experimentation, the results of the training fold were considered acceptable. The trained model was run against the evaluation fold, and we reported these results as an estimation of how the model would perform on an independent dataset, which was not seen in model training.

The standard but naive approach would be to shuffle all the frames together into these datasets, divided so that each class belonged in weighted proportion to each fold. This works for many machine learning problems, but not this one. Suppose in video A, frame #3 belongs to the training fold, and frame #4 belongs to the evaluation fold. As those two frames are substantially similar in their visual content and likely have identical labels, this would create a false impression of the true performance of our model: the model might simply memorize frame #3 and report its label rather than make any difficult determinations. An additional challenge is that the dataset’s videos are of varying lengths and do not feature a similar distribution of activities or intensities, so we cannot simply select random videos. Instead, we calculated the class distribution of all 79,507 possible video-fold assignments and compared the actual distribution of classes in the folds to the overall class distribution in the dataset. We then selected the distribution that has the smallest χ2 Distance [33], subject to a few additional constraints. First, we ensured that our splits had one example of each class in every fold possible, and second, we targeted the 80%/10%/10% fold splits. χ2 distance is a statistical measure of goodness of fit and is an effective measurement to compare the difference between a randomly sampled group and the overall population. The video-fold assignment selected is where the weighted distance of all three folds is minimized subject to the constraints used. This resulted in the class distributions shown in Table 1 for each of the taxonomies. Note that even though the distribution of videos in each taxonomy’s respective folds happened to be very similar across all three taxonomies, this was just a coincidence: there is no requirement that a video needs to be in the same or different folds taxonomy-to-taxonomy. Note that because we only had two videos that depicted running, it was impossible to create a fold that satisfied the three-fold class constraint for running: for all metrics involving Taxonomy 2′s running, we report the testing score rather than the evaluation score.

### 2.3. Model Selection

We developed four specialized machine learning models and applied them to each of the three taxonomies. The models are based on analysis of a single still image from a video. In our search for a baseline, we considered several prior studies that presented successful results when using machine learning techniques for physical activity detection [18,34,35]; however, none of these studies used the taxonomies we selected, which are aligned with standard definitions in physical activity and health research. Additionally, to evaluate the performance obtained from training the four models discussed in this paper, we considered a fifth model trained on one of the taxonomies (Taxonomy 1: sedentary vs. non-sedentary) as a benchmark to which the performance of other models for that taxonomy can be compared.

The four machine learning models that were evaluated for each taxonomy, three deep and one linear, are ResNet with Split Attention (ResNetSt) [36], Vision Transformer (ViT) [37], Convolutional Vision Transformer (CvT) [38], and XGBoost [39]. The first three are pre-trained deep learning models, which were fine-tuned on our dataset, while the XGBoost was the sole linear model we studied. The fine-tuning process, traditionally referred to as transfer learning, leveraged pre-trained open-source deep learning models that are provided by large tech companies that are trained on general object detection datasets. For each transfer learning exercise, we removed the final output layer of the pre-trained model, which does not correspond to our new target taxonomy labels. We replaced that output layer with an uninitialized output layer that was optimized on our own dataset and contained our target taxonomy labels (e.g., sedentary or non-sedentary). We then performed classic machine learning training, only adjusting the parameters in the output layer, leaving all the rest of the pre-trained architecture unmodified. Each model had slightly different expected input, but they all worked on raw video frames that had been cropped, resized, and normalized to a specific value range. In addition, we augmented our training data by randomly modifying it each epoch: randomly modifying lightness values (+/−10%), flipping the image horizontally, or randomly cropping within the frame itself. The purpose of this was to prevent the model from overfitting on many similar frames—particularly on sedentary examples where the subject and camera frame were relatively unchanged for long periods of time—and to allow the model to be more tolerant of different quality video or video perspectives. The utility of this technique has been thoroughly documented [40].

Our fifth benchmark Machine Learning Model is a simple ResNet50 convolutional neural network architecture [41] trained fully and completely from scratch on the images from the dataset used in this study and only on those. Since it was introduced in 2016 [41], ResNet50 has become one of the most popular CNN architectures for the analysis of still images. Trained fully and solely on the data from our study, this model serves as a good benchmark because it is sufficiently sophistcated, as it uses a well-known and successful convolutional neural network architecture. We use the benchmark model to illustrate the difficulty of learning the taxonomies studied in this paper. The benchmark model was trained for Taxonomy 1 (sedentary/non-sedentary). After reviewing its performance, we decided to skip training this benchmark model on the more complex Taxonomies 2 and 3 in order to save computing time—Section 3 discusses this decision in more detail.

Each model was trained three times using recommended parameters referenced from their respective source papers and evaluated on our testing fold after each epoch of the training fold. We selected the best epoch which had the best testing fold score on all three runs for each model, running as many epochs as required until we had 10 epochs without showing any improvement. For ViT, unexpectedly, the very first epoch performed the best, as it started to rapidly overfit the dataset. The other two models saw a more typical number of epochs before starting to overfit (5–10 epochs). Below is a very brief description of each model:

ResNetSt [36] was trained on Common Objects in Context (CoCo) [42], which contains 330k images with over 1.5 million tagged images and 250k tagged people. Based on the classic ResNet50 model [41], ResNet50 uses a chain of many convolutional layers, which allow the model to first identify simple shapes such as lines vs. circles and then to combine those into more complex shapes such as a nose or a cup and then finally learn the context around those objects, such as a face or a table. ResNetSt adds an Attention model, which effectively allows those earlier simple details to be forwarded to future layers rather than dropped entirely. The “Split” Attention has to do with only some of those said features being forwarded.

ViT [37]: This model was trained on ImageNet-21k [43], which has 14 million images with 21k different types of objects. The Transformer Architecture has become the current state of the art for Natural Language Processing (NLP) Taxonomies and has only recently been applied to vision taxonomies. ViT uses the NLP Transformer model by treating patches of 16 × 16 pixel data as something analogous to a “word”. Rather than sentences or paragraphs, we have a picture that is simply a composition of many of these visual “words”.

CvT [38]: Similarly to ViT, this model was trained on ImageNet-21k [43]. The ViT Architecture has a strict ordering of its words, which makes sense as grammar and semantics matter tremendously for conveying information in NLP taxonomies. However, when applied to pictures, the exact order of color patches does not really matter. A slight tilt of a camera, taking the picture in shadow, or capturing some leaves twisting in the background of the frame rarely factually changes the content of the image from a taxonomical perspective. To make the input less sensitive to these small perturbations, CvT re-introduces convolutional patches, which breaks the strict order-dependence of color patches as well as makes the model smaller, giving us the opportunity to train a higher number of epochs in the same amount of time as well as reducing the run time of this model when run by our users.

XGBoost [39] is a linear model built around gradient-boosted decision trees: many very small and simple machine learning models that all vote. The final determination is a weighted average of their collective output. The weights vary from class to class and tree to tree. For example, one of the trees may be a “one issue voter” that is very good at detecting a specific type of label in a specific context but has very little predictive power otherwise. The booster will assign a high value to that tree *only* when it detects a label it is good at detecting and more-or-less ignores it otherwise. Other trees may be more generalist voters, and so taking the average of their individual decisions to form a collective decision gives better accuracy than any one individual tree. XGBoost works from tabular data rather than pixel data; therefore, we first converted the frames to high-quality tabular features. We used AlphaPose [44] as a skeletal-extractor model, which detected people in the frames and output the location and orientation of their limbs. Since XGBoost is a linear model, it trains extremely quickly, giving us the ability to do a high number of epochs and thoroughly experiment with different parameter combinations.

ResNet50 [41] (our benchmark model) is a deep convolutional neural network model in which every three convolutional layers in a row are organized in the form of a residual network, i.e., a neural network (block) designed to predict the residual between the learned function *F*(*x*) and the input *x.* He et al. showed that a residual network architecture learns faster and more accurately than a regular CNN [41]. ResNet50 works well as our benchmark model. It is a well-known neural network architecture that has been proven to be accurate in many settings and is compatible with image recognition. At the same time, to our knowledge, there are no publicly available pre-trained versions of ResNet50 on any of the taxonomies used in our study, giving us the opportunity to train a ResNet50 model from scratch using only the still frames from our dataset. Our benchmark model uses a freshly initialized 50-layer ResNet architecture with a learning rate of 0.001, trained for ten epochs, which are industry standard starting points for a ResNet architecture. We evaluated the model after each epoch against the test fold, saving a copy of its parameter weights. We then selected the copy that performed best on the test fold and evaluated it to our evaluation fold.

### 2.4. Statistical Evaluation

The Area Under the Receiver-Operator-Characteristic Curve (AUC) was used as the primary metric for selecting the final model. An AUC of 1.0 represents perfect discrimination, and 0.5 represents chance discrimination. The AUC visualizes and quantifies the trade-off between too many false positives and too many false negatives. The AUC works well with imbalanced class distributions. We defined the “optimal” decision point as simply the one with the fewest cumulative sum of false positives and false negatives for each classification taxonomy. By default, the AUC only works on the binary decision taxonomy. To convert it to our taxonomies with more than two classes, we used a technique called “One Versus Rest”. We broke the problem into multiple subproblems considering only one class at a time, which were called the “positive class”. We created the Receiver-Operator-Characteristic Curve for each positive class and summed up all the other class predictions as just one generic negative class. We then took the average of all these sub-AUCs to get our averaged AUC. This technique made the evaluation of each class in a taxonomy an independent affair and allowed us to substitute the results of detecting the “running” class in Taxonomy 2 in the evaluation dataset with the results from the test dataset without affecting all other results.

For the evaluation fold, we present confusion matrices with our frame-by-frame hand-annotated ground truth compared to the predicted class. A perfectly accurate model would only have numbers along the diagonal, and non-diagonal values indicate errors. To help summarize each table, we also calculated the precision and recall. Precision ((true positives/(true positive + false positive)) is a measure of how often the predicted value is correct for a *specific* class. Recall (true positive/(true positive + false negative) is the percentage of examples in a specific class that were correctly detected. High precision but low recall indicates that the model has a high false negative rate, but when it does report a class, it is almost always correct. Low precision but high recall indicates that the model is oversensitive and has a high false positive rate. This can also be common in imbalanced problems; suppose you have 10,000 examples of Class A but only 100 examples of Class B. You correctly identify every Class B, but you incorrectly identify 1% of Class A as Class B. Class A was much more common, and 1% of its examples were equal to the total amount of Class B instances. Therefore, in this example, it would have 100% Precision and 99% Recall for Class A, 50% Precision and 100% Recall for Class B, and a total accuracy of 99%. We also calculated the F1 statistic, which is a weighted harmonic mean of precision and recall (2 × [precision × recall]/[precision + recall]). The F1 provides an overall estimate, particularly for situations with an uneven class distribution. The F1 metric takes values between 0 and 1, with values closer to 1 being better and a value of exactly one corresponding to perfect precision and recall. For multiple class problems, we report the weighted precisions, accuracies, and F1 scores. As previously mentioned, since we only had two videos that depicted running, it was impossible to create a fold that satisfied the three-fold class constraint for running: for all metrics involving Taxonomy 2′s running, we report the testing score rather than the evaluation score. All analyses were done in Python, using JupyterLab as our primary visualization tool.

## 3. Results

### 3.1. Deep Learning Model Comparison and Selection

The results of the evaluation of each model are summarized in Table 2. Overall, ViT performance was consistent across taxonomies; however, it is the largest model requiring 13 GB of vRAM. XGBoost was by far the fastest model to train once AlphaPose’s feature extraction has been completed and does acceptable on Taxonomy 1 and Taxonomy 3 (where it performs the best), but on Taxonomy 2 it underperforms both Transformer models. However, AlphaPose extraction is approximately 3:1, making this method overall slower (0.3 frames per sec [fps]) (video length to wall time). ResNetSt did not perform well on Taxonomies 2 and 3, was slightly behind the other three models on Taxonomy 1, and was approximately eight times slower than CVT (8 fps). CVT performed best on the simplest taxonomy and was within 2% of the best-performing models or the other two taxonomies, and it is comparatively fast during both training and inference (64 fps), making it a compelling option as a single model to be deployed on off-the-shelf computers. The final model selection was based on the AUCs, as this showcases approximately how reliable signals each classifier is loading from compared to random chance and can be adapted to work well for multiclass problems. For Taxonomy 1 (sedentary vs. non-sedentary) the final model selected was CvT (AUC = 92.1%, which is a 41.1% lift over the benchmark). For Taxonomy 2 (Activity type), the final model selected was ViT (AUC = 72.3%). For Taxonomy 3 (Activity Intensity) the final model selected was XGB (AUC = 73.4%).

### 3.2. Taxonomy-Specific Model Performance 

To break down the performance of each model on a class-by-class basis, we show confusion matrices in Table 3 for the final model selected for each taxonomy, along with class-specific precision and recall (Table 4).

Taxonomy 1 (Sedentary vs. Non-sedentary) was the simplest taxonomy, and overall performance was high, with an overall AUC of 92.1 and 87.4% accuracy. Weighed precision was 0.88, recall was 0.91, and F1 score was 0.87 (Table 4). There was a slight bias toward the active class despite it being the minority class.

Taxonomy 2 was the activity type, and performance for this four-class model was lower than Taxonomy 1, with an overall AUC of 72.3 and accuracy of 63.1% (Table 4). Similar to Taxonomy 1, there were misclassification errors where we classified sedentary activity as active or active as sedentary (Table 3). Differentiation between mixed movement versus walking was strong (walking was never misclassified as mixed movement, though about 26% of mixed movement does get misclassified as walking). Running, however, often gets misclassified as the other movement classes, though that is not surprising given its small class representation (1.8% of the testing dataset). Weighted precision was 0.73, recall was 0.63, and F1 was 0.65 (Table 4).

For Taxonomy 3 (activity intensity), model performance was 73.4 overall AUC and an overall accuracy of 68.6%. Weighted precision was 0.87, recall was 0.68, and F1 score was 0.75 (Table 4). There was a mostly natural error curve where egregious errors (mistaking vigorous as sedentary or vice-versa) are rare, but misclassifying as an adjacent class happens more often. We note a similar pattern as Taxonomy 2 where we tend to confuse vigorous activity (such as running) with more moderate activity (such as walking; Table 3).

Table 2 and Table 4 also show the performance of the benchmark model (ResNet50 trained only on our dataset and not pre-trained on any external datasets). On the two-class sedentary-non-sedentary taxonomy, the benchmark model exhibited a 56.25% validation accuracy, which is only slightly better than random. The micro-averaged f1 score was 0.53; as this was a binary classifier, its precision and recall were also 0.53. The AUC metric (Table 2) was 51%—not much better than random choice. After observing these results, we elected not to proceed with training the benchmark model on the remaining two taxonomies, as it is clear from comparing the benchmark model to our four other models on Taxonomy 1, the simplest to detect taxonomy, that the benchmark is severely underperforming, and is doing barely better than random choice. In our experiments, which typically consider per-class and multiclass values, these values will differ. Of note, the specialized models performed significantly better for the same task.

## 4. Discussion

This study successfully uses deep learning models trained on continuously recorded third-person videos to predict details about body posture, activity type, and intensity of physical behavior using ground-truth classifications relevant to physical activity and health [22,24]. This is the first known study to apply machine learning methods for computer vision to consensus labels in physical activity research, including the Compendium of Physical Activities and Sedentary Behavior Research Network Consensus Terminology Project. We found a simple classification of sedentary versus non-sedentary had high accuracy (87%) that was comparable to the inter-rater agreements for the gold-standard of human annotation [13,26]. As the complexity of the classification problem increased (i.e., to four activity types and four intensity categories), the overall accuracy declined, but overall, this proof-of-concept application of computer vision to automatically annotate video-recorded direct observation is promising and warrants future research to examine methods to improve accuracy.

The results from the present study are comparable to those of Carlson et al. [26], who used computer vision to estimate measures of a direct observation tool System for Observing Play and Recreation in Communities (SOPARC), which is a momentary direct observation system that includes periodic environmental scans of a particular setting (e.g., park) and documenting how many people are in the setting at a given time and the current activity level (sedentary, walking, or vigorous) [45,46,47]. They found concordance correlation coefficients that were considered moderate to good, ranging from 0.55 for the number active in a scene to 0.88 for the number of people in the scene, as compared to manual annotation [26]. Renzai et al. estimated sit-to-stand transitions and sitting, standing, and walking in a sample of Parkinson’s Disease patients completing scripted taxonomies in a laboratory setting. They reported an overall weighted accuracy of 84.0% in their test fold [34]. In the present study, under less controlled conditions and more detailed aspects of physical behavior, we observed accuracies that align with previous studies, with results ranging from 63% to 87%.

Other applications of computer vision in physical activity and health research have focused on quantifying aspects of the built environment that are important for physical activity promotion [48]. Adams et al. found that a deep learning approach applied to Google Street View was able to classify microscale features of pedestrian streetscapes (e.g., sidewalks, walk signals) with >84% accuracy compared to human annotation [25]. Yue et al. [49] also applied convolutional neural networks to Google Street View and found validation accuracy of >82% for environmental features and subsequently linked those predicted built environment features with chronic conditions and mental health [48]. Given the diminishing cost of image/video data collection and storage, image-based analyses may provide important contextual information for obesity-related research, including nutrition and physical activity [48]. Cameras have been used to quantify the extent to which an environment is “obesogenic” [50], objectively assess travel patterns [51], active transport to school policies [52], and conduct environmental audits of the built environment [28]. Additionally, computer vision has been used to count repetitions in body-weight training exercises (i.e., squatting, burpees, push-ups, sit-ups, and jumping jacks) with high accuracy (>85%) [35]. Collectively, the present study and the previous research suggest a need to continue to develop machine learning methods to automatically annotate images/videos. To date, advancement in algorithm development for wearable devices to predict physical behavior has been hindered by a lack of commonly labeled data and the excessive cost of collecting and manually training direct observation data. The potential utility of computer vision to automate aspects of data annotation would dramatically increase the feasibility, scale, and efficiencies of future research in this area.

Strengths of this study include a protocol that included third-person image data and ground-truth measures that are labeled consistent with consensus terminology in the field of physical behavior assessment [22,23,24]. Previous work in this area has focused on first-person or “ego-centric” cameras or video recordings, which have many strengths but provide less detail on specific body posture and movements (e.g., steps) that may be important for algorithm development metrics like steps or body posture that are needed to train wearable sensor algorithms [28,29,30,51,52,53,54,55,56]. Limitations include data from one geographical region and, although efforts were made to ensure a range of activities, the class distributions were unbalanced. Future research should collect additional data to ensure more balanced distributions and that more detailed taxonomies can be evaluated. The ground truth is human-labeled data, and although the inter-rater reliability was high (ICC = 0.95), there is the possibility of human errors or inconsistencies. This work was conducted on data collected with the explicit permission of the study subjects and for the purpose of analyzing similarly collected videos generated and used in research contexts with collection and use protocols approved by the respective Institutional Review Boards (IRBs). There are established ethical frameworks for the collection of camera data in research contexts [57], but this type of research is not without risk [58], and we advise that future use of these and derivative modeling techniques should always be subject to the appropriate rules and regulations and IRB approval.

There are several clear avenues of improvement: first and foremost, collecting additional annotated data will enhance the robustness and generalizability of our results. We attribute at least part of the errors we reported to the underrepresentation of certain classes in our training data (see Table 1). Additional data collection is currently underway and involves videos from multiple research groups. This additional data collection will allow us to diversify the training data both in terms of physical activity representation but also in terms of the demographics of the subjects and the variety of contexts in which they are filmed (observations conducted in coastal California are unable to represent snow as the setting, for example). Future research will consider refinement of feature selection. Our current work uses image pixels and AlphaPose “stick figure” representations of the subject in the video as the only types of features. We are planning to explore additional pose detection feature extraction options, such as Google’s MediaPipe [59]. Other feature extensions discussed below will involve the use of more than one still frame per observation. There are several improvements to the machine learning models that we are considering. First, for the more complex classification in Taxonomies 2 and 3 (activity type and intensity), merging the AlphaPose features into the Vision Transformers as a secondary input seems promising and has been suggested in similar video analysis problems [60]. Second, we are planning to investigate the effect of analyzing short video fragments instead of individual still images. This will entail making the input recurrent: that is, rather than only using a single frame, we plan to use a collection of frames: for example, the previous 4 s and 1 s into the future (if used in a non-real-time application). This could be done by using a (i) fixed convolution on the input being fed into the existing pipelines, or (ii) using a ConvLSTM [61] model that was designed for pooling together sequences of similar images that are taken at different time stamps, or (iii) using a ViViT [60] which is a Video Vision Transformer with a single label, or (iv) having a sequence of convolutional layers in addition to the existing Transformer models that are eventually merged into the final output layer. With more information available, models trained on short video fragments may show improved accuracy, potentially at the cost of runtime performance (both for training and for using a trained model to annotate a full video), as such models ingest significantly more input. Finally, with multiple trained models that use different inputs, we plan to investigate a variety of ensemble techniques to see if we can improve model accuracy by using multiple classifiers (we note parenthetically that some of our methods like XGBoost can already be considered a form of an ensemble classifier, but in this particular case, we are talking about combining results of multiple different trained models).

There are many advantages to using computer vision to label video recordings of physical behavior, including increased scalability and potentially more consistency compared to manual annotation by human observers. The present study was the first to apply computer vision to established taxonomies in physical behavior research within naturalistic environments. Although the accuracy for Taxonomy 1 (Sedentary/Non-sedentary) was comparable to human annotation, future research that includes additional model development and validation on larger and more diverse datasets is needed to enhance the accuracy and generalizability of the models for the more complex taxonomies. For optimal use of computer visions as ground-truth measures, researchers will likely need some way to evaluate the performance of the machine learning models on a frame-by-frame basis and the ability for researchers to “correct” errors in the predictions, such as within an annotation software. This application of computer vision to classify aspects of posture, activity type, and intensity from a third-person video demonstrates a promising proof of concept that should be explored in future research.

## Figures and Tables

**Table 1:** Frame Class Distribution by Taxonomy and Fold.

**Taxonomy 1: Sedentary**					
**Fold**	**# Vid**	**Sedentary**	**Active**		
Training	32	116,759	111,184		
Testing	6	19,329	16,816		
Evaluation	5	14,618	14,994		
**Taxonomy 2: Activity**					
**Fold**	**# Videos**	**Sedentary**	**Mixed movement**	**Walking**	**Running**
Training	32	69,858	13,206	34,884	4090
Testing	6	1848	731	687	5106
Evaluation	5	2627	618	2099	0
**Taxonomy 3: Intensity**					
**Fold**	**# Videos**	**Sedentary**	**Light**	**Moderate**	**Vigorous**
Training	32	125,363	70,081	24,707	7551
Testing	6	15,325	13,427	1752	5228
Evaluation	5	10,009	18,519	1453	267

**Table 2:** Comparison of Area Under the Curve for Four Machine Learning Models by Taxonomy.

Taxonomy	ResNet50	RNSt	ViT	CvT	XGB
1: Sedentary vs. non-sedentary	51.0	88.9	90.1	**92.1**	90.8
2: Activity type	N/A	47.4	**72.3**	71.4	65.8
3: Activity intensity	N/A	71.6	70.1	71.9	**73.4**

Note: Bold font indicates best performing model for each taxonomy. ResNet50 is the benchmark model that was only applied to taxonomy 1, so results are N/A for other taxonomies.

**Table 3:** Confusion Matrices by Taxonomy for Evaluation Phase.

**Taxonomy 1: Sedentary vs. Non-Sedentary**							
**Label\Predicted**	**Sedentary**	**Active**			**Precision**	**Recall**	**F1**
Sedentary	**11,926**	2692			0.92	0.82	0.87
Active	1026	**13,968**			0.84	0.93	0.88
**Taxonomy 2: Activity Type**							
**Label\Predicted**	**Sedentary**	**Mixed** **Movement**	**Walking**	**Running**	**Precision**	**Recall**	**F1**
**Sedentary**	**2338**	0	283	6	0.65	0.89	0.75
**Mixed movement**	307	**758**	381	7	0.53	0.52	0.53
**Walking**	928	0	**1166**	5	0.34	0.56	0.42
**Running**	2	663	1580	**2861**	0.99	0.56	0.72
**Taxonomy 3: Activity Intensity**							
**Label\Predicted**	**Sedentary**	**Light**	**Moderate**	**Vigorous**	**Precision**	**Recall**	**F1**
**Sedentary**	**13,259**	4915	323	22	0.90	0.72	0.80
**Light**	197	**939**	115	14	0.11	0.74	0.20
**Moderate**	1253	2344	**6352**	60	0.92	0.63	0.75
**Vigorous**	2	83	110	**72**	0.43	0.27	0.33

Note: Values are video frames. The running results come from our testing fold and are heavily overweighted, making up 49% of the confusion matrix values despite being a class weight of only 3% of the total dataset. Therefore, we have suppressed the running AUC weight by 94%.

**Table 4:** Weighted Average Results for Each Taxonomy for Best-Performing Models.

	Precision	Recall	F1	Accuracy
Taxonomy 1: Sedentary/not: Benchmark model	0.50	0.66	0.31	56.3%
Taxonomy 1: Sedentary/not	0.88	0.91	0.87	87.4%
Taxonomy 2: Activity type	0.73	0.63	0.65	63.1%
Taxonomy 3: Activity intensity	0.87	0.69	0.75	68.6%

Note: The benchmark model was ResNet50 and was trained only on our images. The results for the other three taxonomies were the best-performing of the four trained models. For Taxonomy 1, this was CvT. For Taxonomy 2, this was ViT. For Taxonomy 3, this was XGB.

## Data Availability

Due to the participant being identifiable in the videos, a limited subset of data presented in this study are available on request from the corresponding author.
